# Smartphone video nystagmography using convolutional neural networks: ConVNG

**DOI:** 10.1007/s00415-022-11493-1

**Published:** 2022-11-23

**Authors:** Maximilian U. Friedrich, Erich Schneider, Miriam Buerklein, Johannes Taeger, Johannes Hartig, Jens Volkmann, Robert Peach, Daniel Zeller

**Affiliations:** 1 Department of Neurology, University Hospital Wuerzburg, Josef-Schneider Strasse 11, 97080 Würzburg, Germany; 2 Brandenburg University of Technology Cottbus, Senftenberg, Germany; 3 Department of Otorhinolaryngology, University Hospital Wuerzburg, Josef-Schneider Strasse 11, 97080 Würzburg, Germany; 4 Department of Otorhinolaryngology, University Hospital Aachen, Pauwelsstraße 30, 52074 Aachen, Germany; 5 Department of Brain Sciences and EPSRC Centre for Mathematics of Precision Healthcare, Imperial College London, London, SW7 2AZ UK

**Keywords:** Digital medicine, Nystagmus, Eye movement disorders, Videooculography, Computer vision, Telemedicine, Precision medicine

## Abstract

**Background:**

Eye movement abnormalities are commonplace in neurological disorders. However, unaided eye movement assessments lack granularity. Although videooculography (VOG) improves diagnostic accuracy, resource intensiveness precludes its broad use. To bridge this care gap, we here validate a framework for smartphone video-based nystagmography capitalizing on recent computer vision advances.

**Methods:**

A convolutional neural network was fine-tuned for pupil tracking using > 550 annotated frames: ConVNG. In a cross-sectional approach, slow-phase velocity of optokinetic nystagmus was calculated in 10 subjects using ConVNG and VOG. Equivalence of accuracy and precision was assessed using the “two one-sample *t*-test” (TOST) and Bayesian interval-null approaches. ConVNG was systematically compared to OpenFace and MediaPipe as computer vision (CV) benchmarks for gaze estimation.

**Results:**

ConVNG tracking accuracy reached 9–15% of an average pupil diameter. In a fully independent clinical video dataset, ConVNG robustly detected pupil keypoints (median prediction confidence 0.85). SPV measurement accuracy was equivalent to VOG (TOST *p* < 0.017; Bayes factors (BF) > 24). ConVNG, but not MediaPipe, achieved equivalence to VOG in all SPV calculations. Median precision was 0.30°/s for ConVNG, 0.7°/s for MediaPipe and 0.12°/s for VOG. ConVNG precision was significantly higher than MediaPipe in vertical planes, but both algorithms’ precision was inferior to VOG.

**Conclusions:**

ConVNG enables offline smartphone video nystagmography with an accuracy comparable to VOG and significantly higher precision than MediaPipe, a benchmark computer vision application for gaze estimation. This serves as a blueprint for highly accessible tools with potential to accelerate progress toward precise and personalized Medicine.

**Supplementary Information:**

The online version contains supplementary material available at 10.1007/s00415-022-11493-1.

## Background

Disturbances of ocular motility and coordination mark a highly interdisciplinary subject matter extending into the fields of neurology, ophthalmology and otology. Somewhat unique to motor physiology, eye movements can be grouped into a limited set of functional domains which specifically map onto distinct or partly overlapping neural circuits [1]. These involve vestibular and visual afferents as well as neuronal networks encompassing the vast extent of cortex, brainstem and cerebellum. Therefore, most structural and functional pathologies of the peripheral and central nervous system manifest with eye movement disorders, lending to the notion of the eyes being a “window into the brain”.

Among those, *nystagmus (*from greek “*nystázein*”, to doze*)* has historically attracted the attention of clinicians and scientists due to its localizing and predictive value in various physiological and pathological contexts [2]. This is especially true in the context of vertigo and dizziness, which are among the most prevalent and economically challenging conditions world-wide [3, 4].

Over the last century, different approaches to register eye movements and in particular nystagmus have been developed, ranging from early eye photography to invasive (i.e., scleral search coils), electrical (e.g., electrooculography) and most recently, infrared and video-based [i.e., videooculography (VOG) goggles] techniques (for a review, see [1, 5]). Besides documenting the mere presence and three-dimensional direction of nystagmus, the aforementioned methods enable quantification of slow-phase velocity (SPV) as the quintessential descriptor of pathophysiology underlying nystagmus. Traditionally, SPV serves as the prime kinematic descriptor of nystagmus dynamics and is relevant to diagnostic [6, 7] and therapeutic decisions alike [8, 9].

Common to VOG methods, however, is a high degree of resource intensiveness encompassing both monetary and educational aspects, hindering broad use in diverse clinical settings and especially outside of highly specialized laboratories, high-resource settings and academic infrastructure. This can be viewed as a significant care gap, since recent clinical investigations convincingly demonstrate clinically relevant benefits of quantitative eye movement recordings for diagnosis [6, 10–12], prognostication [13] and disease monitoring [9, 14, 15]. Moreover, yet unknown disease patterns could be identified within the granular kinematic feature space provided by, e.g., videooculography, hinting at a largely untapped potential to derive performant physiomarkers to be included in clinical decision making [6, 16].

The episodic and often evanescent nature of many frequent neurological conditions presenting with eye movement abnormalities (e.g., vestibular migraine which affects up to 1% of the population [17]) is associated both with missed diagnoses and misdiagnosis [6, 18]. Therefore, broadly available, point-of-care and longitudinal monitoring solutions to capture acute vertigo and improve diagnostic accuracy have received increasing attention [7, 19], with the idea of “telemetric nystagmography” dating back to 1991 [20].

Undoubtedly, the introduction of deep learning (DL) and computer vision (CV) in the neurosciences has had a profound impact on the way behavior is measured in experimental [21, 22] contexts. With the introduction of DeepLabCut (DLC) [23] (and comparable tools, see [24] for a review), which is a highly accessible supervised deep learning framework extensively validated across several species [25], markerless pose tracking has now become the state-of-the-art in experimental neurosciences. In recent years, CV techniques have increasingly permeated into the realms of clinical and especially motor neuroscience [26–33]. At the core of these supervised DL frameworks commonly lie convolutional neural networks (CNNs) pretrained on several thousands to a million naturalistic images [34], which are fine-tuned by the user for specific use cases. In contrast, benchmark human pose detection algorithms like OpenFace [35, 36], Google’s MediaPipe [37] or Apple’s augmented reality kit (ARKit) [38], mostly validated with large ground truth datasets (e.g., face and body tracking), promise “out of the box” functionality and real-time tracking without additional user annotation. First clinical applications for face and limb kinematics [39, 40] and proof-of-concept studies investigating eye and head kinematics [41, 42] yielded potentially clinically translatable results. However, to date, it is unknown whether popular CV frameworks are sufficiently robust for clinical applications like nystagmus assessment.

Given the worldwide practiced tradition of clinical photo- and videography, especially in the field of Neurology and Ophthalmology, CV has the potential to unlock numerous opportunities for data collection, likely containing digital biomarkers for prediction, prevention, prognostication and diagnostics [31, 32, 43].

Here, we set out to develop a robust yet simple to use framework based on DLC to investigate whether SPV can be calculated from smartphone nystagmus videos taken in naturalistic, clinically relevant scenarios, both prospectively in healthy controls and retrospectively in clinical cases. We validate these measures against the current clinical gold standard, infrared VOG as well as existing computer vision benchmarks for gaze estimation.

## Methods

**Fig. 1:**
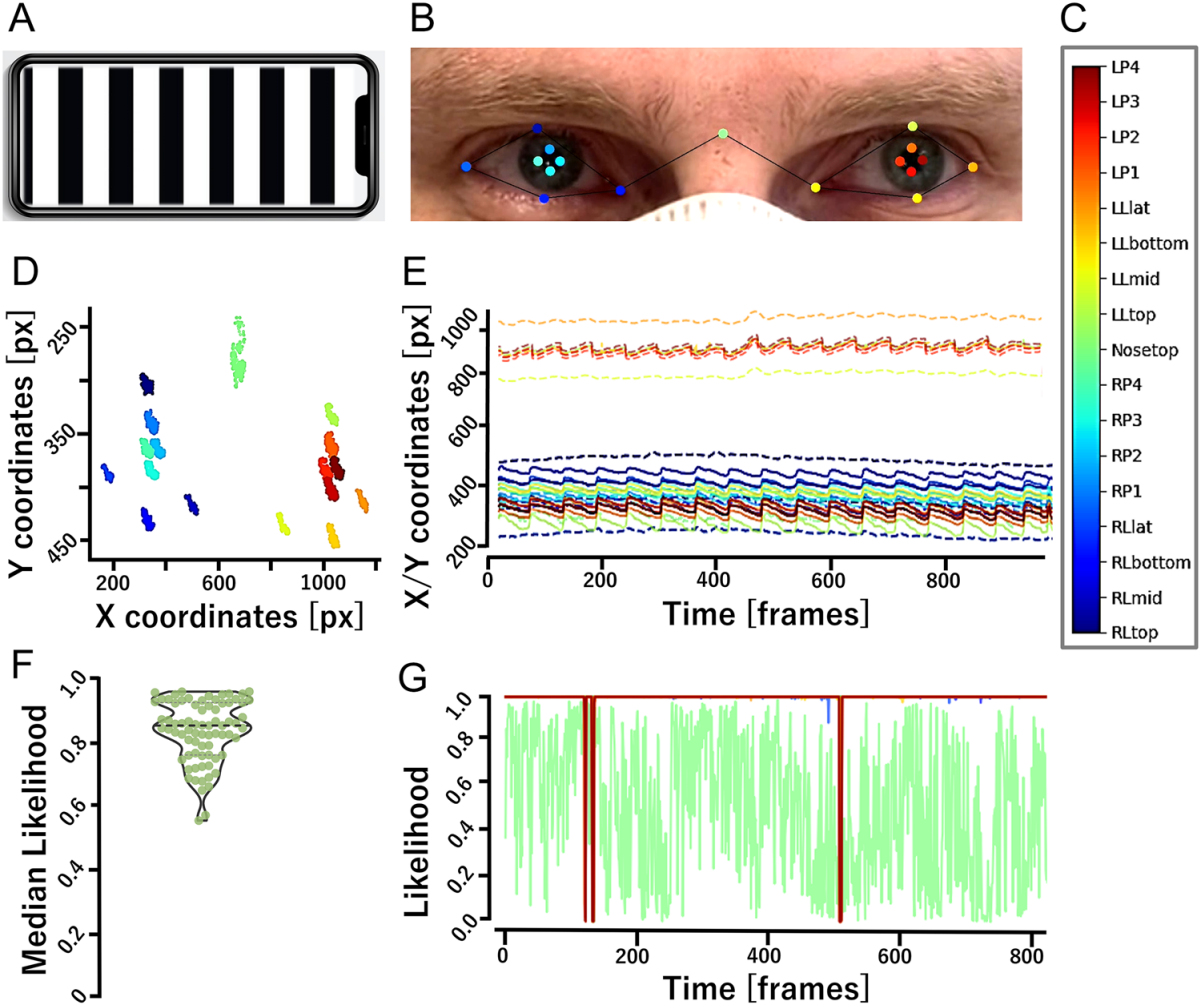
Workflow illustration. **A** Standardized optokinetic stimulus presented on a smartphone screen. **B** Exemplary frame of a tracked video, showing the typical camera perspective as well as ConVNG marker predictions, the color code of which is shown in (C). *L* left, *R* right, *P* pupil, *L* lid, *lat* lateral, 1–4 denote pupil marker position where 1 and 3 represent top and bottom (clock position 12 and 6) and 2 and 4 medial and lateral, e.g., “LP3” denotes left pupil bottom marker and “RLmid” the medial border of the right eyelids etc. **D** Exemplary raw aggregate data plot and **E** raw coordinate time-series plot from ConVNG-derived landmarks. Upper portion depicts vertical coordinates, lower portion horizontal coordinates. Note the already clearly recognizable “saw tooth” appearance typical for nystagmus in **E**. **F** Median likelihood of predicted pupil labels in the out-of-sample validation approach using 10 independent videos. **G** Exemplary likelihood plot from all landmarks derived of the same video as B. Except for “nosetop” marker, all landmarks are tracked with constantly high likelihoods (~ 1.0)

See Fig. 1 for an illustration of the methodological workflow.

### Tuning a convolutional neural network to perform pupil tracking

Utilizing the open-source framework DLC [25], a convolutional neural network (CNN) based on a residual network architecture (ResNet 50) was trained to track a total of 17 landmarks delineating each pupil´s outlines at clock positions 3, 6, 9 and 12 (*n* = 8) as well as anatomically defined support points of interest in the face (eye lid borders, root of the nose, *n* = 9, Fig. 1B). To train a maximally robust model, a total of 558 frames were extracted from > 50 highly variable videos depicting ~ 40 individuals (7–15 frames per individual). The videos were collected from the authors’ own or openly available video collections [44] and showed eye movements and faces of diverse persons in various situations, lightings and camera settings, both in physiological and pathological contexts. To ensure broad coverage of possible pose patterns, the k-means algorithm implemented in DLC [23] was selected for extraction of frames which were subsequently labeled by an expert annotator (MF). In order to exclude labeling errors which may negatively affect generalization, the labeled frames were plotted and quality-checked for accuracy and plausibility before the CNN was trained on a 95% fraction of data leaving the remainder as a test set for later performance evaluation. No videos of the prospective cohort recruited to validate SPV measurements were included in this training dataset to ascertain clear separation between training, test and validation datasets throughout. The CNN was fine-tuned using DLC’s default augmentation and ResNet 50 initialization weights. Sufficient convergence of the loss function was ensured with training iterations ranging between 350,000 and 750,000 in a total of 14 consecutive refinement iterations using unseen video material. Model performance was evaluated using a polypragmatic approach: first, by computing the mean Euclidean distance (MED) of user-annotated and CNN-predicted labels [25], second, by relating the sizes of MED and tracked region of interest as previously reported by our group [45] and third, by systematically inspecting pupil marker likelihoods as a surrogate for the model’s prediction confidence in a fully independent (i.e., out-of-training) dataset of another 10 videos randomly sampled from an open-source, comprehensive eye movement disorder education library (courtesy Dan Gold) [44]. The videos were recorded in real-world clinical contexts, depicting patients with vertigo and eye movement disorders. The videos were taken without any constraints of lighting, camera hardware or viewing distance, thereby intended to best reflect variability encountered in clinical practice.

### Eye movement recordings

#### A priori power analysis

Before subject recruitment, we carried out an a priori power calculation for two (paired) one-sided *t*-tests (TOST) powered to detect equivalence of SPV measurements. Assuming a β/α-ratio of 1.0 and a moderate effect size (*d* = 0.65) due to the multivariate technical superiority of VOG over smartphone video (infrared vs. RGB sensor for contrast maximization, 220 Hz vs. 30 Hz temporal resolution, goggle/head vs. tripod mounted camera), at least 10 subjects were needed for a power > 0.8. This cohort size matches well with comparable studies [38, 42, 46].

### Determination of intervals for equivalence testing

A smallest effect size of interest (SESOI) [47] between 2 and 3°/s (i.e., 2.5°/s) was established in a consensus of the authors specializing in neurotological and neuroophthalmological conditions. This value was inferred so as to reflect a conservative estimate of a minimal SPV sufficient to generate perceptible oscillopsia in humans and to prompt further clinical workup, e.g., when encountered in screening for oculomotor and vestibular disorders, therefore, having theoretical and practical relevance. Of note, SPVs higher than 5°/s are associated with a relevant interference with reading ability [48], which is in our experience a rather liberal estimate. Also to improve statistical rigor, we decided to keep the more conservative value of 2.5°/s agreed upon in said consensus. Corresponding to approximately 25% of the estimated ground truth SPV, this value also matches with clinically used cutoffs to determine directional asymmetry of, e.g., caloric nystagmus [49]. For relative error comparisons, a corridor of accepted measurement deviation was deducted from the deviation extremes measured with gold-standard VOG after outlier testing in order to reduce the chance of misinterpreting an outlier as an acceptable deviation margin. These inferences were based on established methods to determine minimal meaningful effects in clinical studies [47, 50] and are in line with heuristic frameworks of SESOI definition [51, 52].

### Subjects and ethics approval

For prospective validation, 10 healthy subjects (5 female, 5 male) aged 25–44 years without significant neuroophthalmologic abnormalities, normal binocularity and an uncorrected visual acuity > 0.6 were recruited between 2021 and 2022 at the University Hospital Wuerzburg. For the retrospective validation, cases with both VOG and frontoparallel eye movement smartphone video recording available from the same session were screened in the authors’ databases and collections. This query yielded two more subjects who had presented with downbeat nystagmus (DBN, female, 36 years) and congenital nystagmus (CN, male, 55 years) to the lead author’s clinic between 2018 and 2019. In both instances, 30 Hz smartphone camera recordings were collected in an a priori unstandardized fashion for nystagmus documentation purpose. In case of DBN, the smartphone camera was held in the examiner’s hand, whereas in the case of CN, a tripod was used. In both instances, viewing distance was approximately 40 cm. Ethics approval was obtained from Julius-Maximilians University Wuerzburg’s ethics committee under the number 318/21.

### Experimental setup

Experimental procedures were conducted in a naturally illuminated room at daytime. Subjects were seated in a distance of 40 cm in front of a smartphone mounted on a standard tripod with the screen facing frontoparallel. Screen orientation was switched 90° depending on the respective stimulus plane, i.e., using landscape mode for horizontal stimuli. Alignment of the screen with the horizontal and vertical axes was ascertained using a built-in level of the tripod. The smartphone screen’s width was measured to be 14.0 cm, mapping onto 896 *points*, an abstract unit referencing the screen as a coordinate system used by Apple (https://developer.apple.com). This in turn results in a total angular viewing range of approximately 20°, a value which was chosen in accordance with standard VOG paradigms [53]. The height of the tripod was adjusted to align the screen’s center with the meridian of the subject’s eyes. The subjects were instructed to rest the back of their heads against a wall behind them or, in case of sitting freely, to hold the head as steady as possible while recording. The subjects’ interpupillary distance was measured with a distance ruler upon fixation of an object positioned to match the experimental approach (~ 40 cm) for later pixel-to-metric unit conversion.

### Video recording

Eye movements were video recorded with smartphone cameras (iPhone XR, Apple, Cupertino, CA, USA) at 1920 × 1080 pixels and a framerate of 30 Hz, and in the case of one subject, 60 Hz.

### Videooculography

For gold-standard monocular VOG recordings, the “EyeSeeCam Sci” (Version 8,108,847, EyeSeeTec, Munich, Germany [54, 55]) was used. The camera’s spatiotemporal resolution is documented to be 188 × 120 px and 220 Hz, yielding a spatial resolution of 0.05°–0.1° translating into an accuracy of approximately 1°. The VOG camera was connected to a Lenovo ThinkPad T470 Core i7 laptop running the proprietary OtoAccess version 1.5 recording software (Interacoustics, Middelfart, Denmark). The goggles’ headband was adjusted for a firm and snug fit. The camera was aligned in subject’s gaze straight ahead to center the reflection of the pupil in the image frame and subsequently calibrated for every individual using the built-in five-point laser grid projected onto a white wall located in one meter distance. Calibration plots were visually inspected by the experimenter (MF) before recordings were started in the software’s default “nystagmus” mode.

### Stimulus material

To elicit nystagmus in a standardized way, we delivered a monochrome optokinetic nystagmus drum stimulus on the smartphone revolving at a constant rate of 555 points/s (equaling 8.7 cm/s) along the screen, corresponding to an angular velocity of ~ 12°/s (Fig. 1A). This speed was chosen (i) on practical grounds as it allowed optimal stimulus pursuit [56, 57] as well as (ii) because it reflects the typical frequency of both physiologic caloric and pathological vestibular nystagmus encountered in daily practice [6, 7, 9, 58]. Based on the assumption of a normal optokinetic response gain of 0.85 for horizontal and 0.80 for vertical stimuli in control subjects below the age of 50 years [57, 59], the ground truth of slow-phase velocity was estimated to be 10.2°/s for horizontal and 9.6°/s for vertical directions.

### Comparative nystagmus recordings

Since VOG goggles significantly obscured facial features essential for CNN-based pupil tracking, nystagmus recordings were carried out in two back-to-back sessions taking place in a randomized order. To minimize intra-individual nystagmus heterogeneity between recording sessions, subjects were instructed to preferably follow fixation targets until they disappeared from the screen before refixation of a remote target on the opposite side of the screen. Nystagmus was recorded over 30 s in each of the four directions: left, right, up and down with breaks of 2 min between conditions for recuperation. All collected data were included for subsequent analyses.

### Deriving kinematic measurements from eye movement videos

#### Preprocessing

A striking inverse relationship of the CNN’s tracking performance, as assessed by stability and plausibility of landmark predictions upon visual inspection of outputs, and spatial resolution of the videos (i.e., better tracking in lower resolutions) was observed. Evidently, 640 × 480 px was the optimal resolution, retaining enough meaningful spatial information for landmark tracking while being associated with the most favorable tracking performance (Fig. 1D, E, G). Therefore, all videos were downsampled to this resolution.

Kinematic analysis was implemented in Python using standard scientific analysis packages (pandas, sklearn, numpy, scipy). Due to the inexact alignment of video and stimulus onset, the DLC output was first filtered for times of interest (the first and last 5 s are cut from the analysis). Next, the data were cleaned using two steps: (1) we removed low likelihood marker data points (*p* < 0.8, likelihood defined by DLC during prediction), (2) we removed impossible coordinates, i.e., where the markers for the pupil lie outside of the area spanned by the eye lid markers (rarely observed artifact). Upon close visual inspection, these preprocessing steps proved widely sufficient to remove all blink related artifacts, leading either to low pupil marker prediction confidence or implausible coordinates. The missing points could then be interpolated using a median filter to smooth the signal. Furthermore, a low pass filter was implemented to remove remaining high frequency components associated with fast movements like blinks. The tracked pupil marker positions (see diagram of markers in Fig. 1B) were averaged for each eye to determine a pupil centroid, before using a bandpass filter (low cut = 0.5 Hz, high cut = 14 Hz), removing both high frequency noise and low frequency large-scale movements (e.g., slow head or camera movements). In case of camera motion interfering with the nystagmus’ dominant frequency band (e.g., in retrospective case DBN, recorded using a handheld smartphone), binocular lid and canthus markers’ average x and y coordinates were regressed from the pupil markers’ signal so as to create a stable reference frame.

For transforming *pixels* into *metric units*, a conversion factor was derived per individual by dividing the actual interpupillary distance (IPD, average of three measurements from mid-pupil to mid-pupil) by the horizontal distance between both ConVNG tracked pupil centroids in pixels (average ~ 60 mm, in line with previous anthropometric data [60]), before conversion to degrees using angle = arctan (*x*/*r*), where r is the radius of the eye (defined as 12 mm, in line with anthropometric data [61]). Using IPD average instead of individual values did not significantly influence SPV calculations. The time series of each pupil (left and right, in degrees) is smoothed using a median filter (ndimage package, window length = 3).

### Classification of nystagmus direction

The direction of the nystagmus (horizontal or vertical) was determined by calculating a fast Fourier transform of the x and y components of the pupil trajectory. If the absolute power is larger in the x-direction, then the nystagmus fast beating component is horizontal, and vice versa for vertical. Since the nystagmus may have a component in both the x–y directions (e.g., due to slightly oblique camera perspective), we computed the magnitude of the combined trajectories of *x* and *y*, $\sqrt{{x}^{2}+{\mathrm{y}}^{2}}$. The resulting trajectories are further processed by performing a linear interpolation (interp, numpy) between the peaks and troughs (peak detection, scipy, prominence = 1) of the nystagmus trajectory, producing a saw tooth signal.

### Calculation of slow-phase velocity

Slow phase velocity is the primary kinematic measure to characterize jerk nystagmus and, therefore, widely used for its diagnostic classification and monitoring alike [2, 6, 7, 9]. Directional SPV comparisons are routinely used to determine relative asymmetries of caloric excitability of either labyrinth [49, 62]. The SPV for each eye is calculated by multiplying the median of the instantaneous gradient (gradient function, numpy) by the sampling frequency of the original video. The sign of the gradient (positive or negative) per plane indicates whether the slow-phase velocity is leftward, rightward, upward or downward.

### Statistical methods

Normality of datasets was examined using Kolmogorov–Smirnov testing and additional inspection of quartile (“*Q*–*Q*”-) plots to inform selection of appropriate display of data distributions as well as parametric or non-parametric analyses. For SPV analyses, binocular measurements resulting from ConVNG were averaged. For relative error calculations, both method’s absolute deviations from the plane-specific estimated ground truth were divided by the observed value. Equivalence was assessed using the TOST method [51] with smallest effect sizes of interest [52] derived as outlined earlier. In addition, the Bayesian interval-null method for equivalence testing was used [63] (see supplementary data). Outliers were removed using the robust regression and outlier removal (ROUT) method with a balanced coefficient *Q* = 1% [64]. For all statistical computations, GraphPad Prism Version 9, JASP Version 0.14.1 [65] and JAMOVI Version 2.2.5.0 [66], both with R plugins [67] were used. Significance level was set at 5% (i.e., *p* < 0.05).

## Results

### ConVNG model evaluation

CNN performance was evaluated in a tripartite approach. First, the mean Euclidean distances of user-annotated and CNN-predicted labels on the training and test data subsets were measured. These were 2.22 and 6.12 pixels, a ratio reflecting acceptable to good generalization [25]. Second, an additional, pragmatic performance evaluation was carried out by relating the magnitude of the MED in the test set to the size of the tracked structure of interest as previously reported by our group [45]: given the average pupil diameters occupying 40–70 pixels throughout the used video material, MED was calculated to correspond to 9–15% of a pupil diameter and, therefore, confirmed to be acceptably small for further predictions (see supplementary videos 1 through 4 for exemplary labeled videos). Lastly, the median likelihood assigned to each of the eight pupil markers in a set of ten fully independent clinical eye movement videos randomly sampled from an open-source eye movement video collection [44] was 0.85 (95% CI [0.82, 0.86], range: 0.2–0.96 before outlier detection and 0.55–0.96 after removal of four outlier values identified using ROUT method at a *Q* = 1%, Fig. 1F), demonstrating high out-of-sample model robustness. Upon further inspection, the lowest likelihood labels were yielded by one video. A lack of visual separability of pupil edge and an exceedingly dark iris could be identified as the most likely confounding factor.

To establish a benchmark, ConVNG’s pupil tracking performance was additionally compared to existing machine learning algorithms usable for offline human gaze estimation, namely OpenFace [35] and mediapipe [37] (supplementary Fig. 1 and see supplementary data).

### Accuracy and precision of slow-phase velocity calculations

As the prime parameter to characterize intensity of jerk nystagmus, VOG-based SPV calculations are routinely used in clinical practice both in diagnostic [6, 7, 62] and therapeutic [9, 14, 68] contexts. To compare ConVNG’s aptitude to determine SPVs of experimentally standardized nystagmus, accuracy and precision were compared to the clinical gold-standard method, infrared VOG.

### Two one-sample *T*-test

**Fig. 2:**
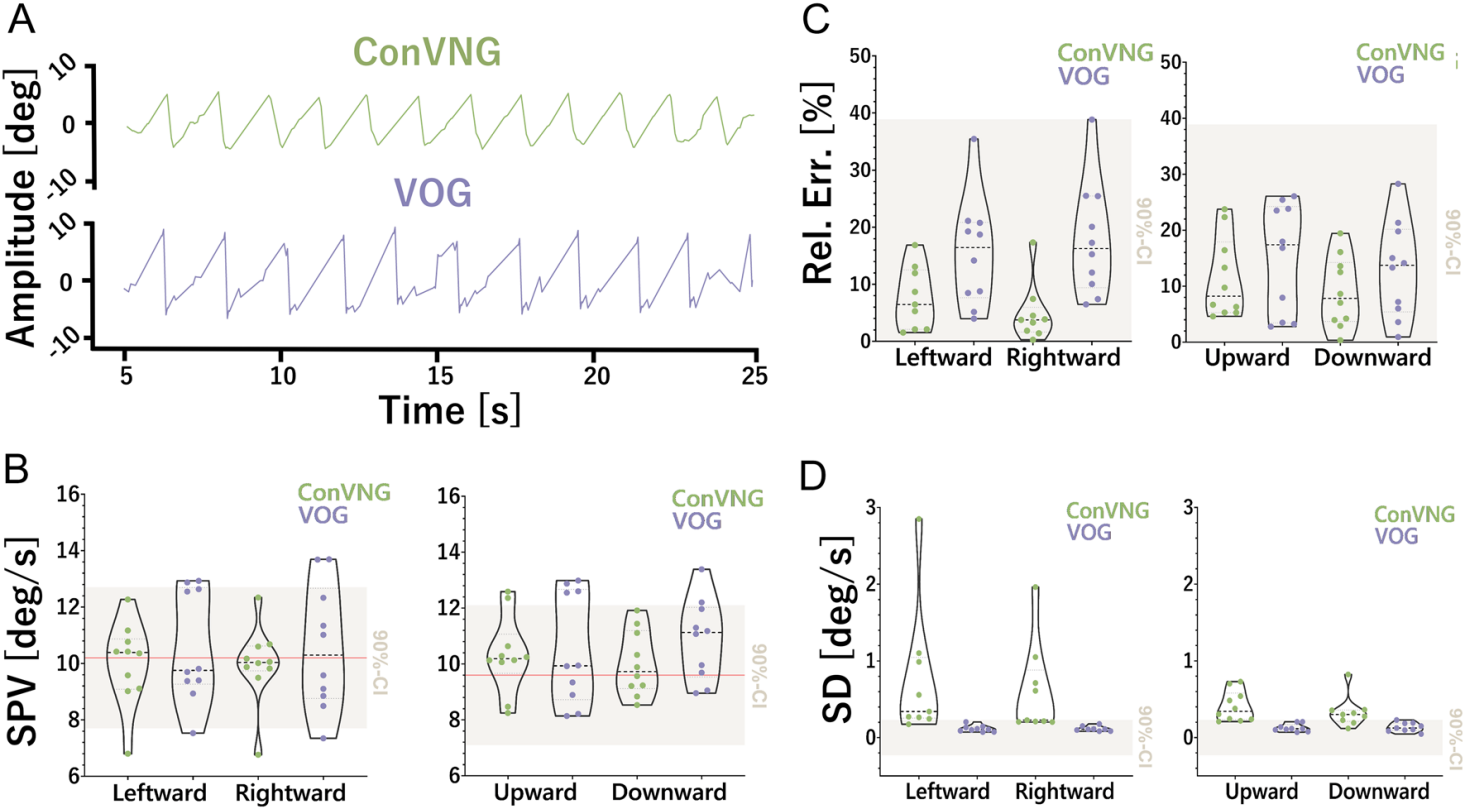
Validation. **A** Exemplary traces derived from ConVNG and VOG for comparison. **B** SPV values for both horizontal and vertical planes are shown in relation to the 90% CI for equivalence testing. Red line denotes ground truth SPV value for horizontal (10.2°/s) and vertical (9.6°/s) planes. ConVNG values fall well within equivalence boundaries (± 2.5°/s). **C** Relative error values for accuracy calculation, in relation to the equivalence interval. ConVNG values fall well within equivalence boundaries (± 38.9%). **D** Standard deviations of sequential SPV measurements per plane for precision calculation, in relation to the equivalence interval. ConVNG values are significantly larger than the upper equivalence boundary, indicating lower precision (means: ConVNG 0.3°/s and VOG 0.12°/s)

Within derived equivalence boundaries of ± 2.5°/s, TOST revealed equivalence of SPV measures in all planes (leftward, upper limit *T*(9) = − 4.5, *p* < 0.001, lower limit *T*(9) = 2.8, *p* = 0.01; rightward, upper limit *T*(9) = − 4.0, *p* = 0.002, lower limit *T*(9) = 2.51, *p* = 0.017; upward, upper limit *T*(9) = − 4.0, *p* = 0.002, lower limit *T*(9) = 3.34, *p* = 0.004; downward, upper limit *T*(9) = − 7.5, *p* < 0.001, lower limit *T*(9) = 3.6, *p* = 0.003, Fig. 2A, B).

To assess directional symmetry, which besides absolute SPV values, is the principle readout for caloric nystagmus assessment in the clinical setting [49, 62], equivalence testing was also performed within methods and between stimulus directions. Per convention, 20–33% of directional asymmetry [49, 62] in the horizontal plane constitutes the cutoff for pathological findings (i.e., caloric paresis). In relation to the ground truth estimate of SPV, these percentages correspond to 2.0–3.4°/s, again rendering ± 2.5°/s a reasonable equivalence interval. Conversely, the assumption of symmetry does not apply to vertical planes in various oculomotor domains [69, 70]. Therefore, only horizontal symmetry was tested. TOST revealed equivalence of directional SPV measures in both methods (ConVNG, upper limit *T*(9) = − 6.5, lower limit *T*(9) = 9.2; VOG, upper limit *T*(9) = − 6.5, lower limit *T*(9) = 6.6, all *p* < 0.001).

The SPV errors per plane in relation to the estimated ground truth were leftward 11.8 ± 14.4, rightward 9.5 ± 15.3, upward 11.4 ± 7.3, downward 8.9 ± 6.3% (median 6.6 ± 11.0%) for ConVNG and 15.6 ± 9.5, 17.8 ± 10, 15.1 ± 9.8, 13.0 ± 8.6% (median 15.1 ± 9.2%) for VOG. For equivalence testing, the maximum deviation of gold-standard VOG (38.9%, survived ROUT at *Q* = 1%) was used as an anchor point for 90% CI assumption, revealing equivalence of methods in all comparisons (leftward, upper limit *T*(9) = – 7.3, lower limit T(9) = 6.02; rightward, upper limit *T*(9) = – 6.8, *p* = 0.001, lower limit T(9) = 4.4; upward, upper limit *T*(9) = – 12.2, *p* < 0.001, lower limit T(9) = 10.1, *p* < 0.001; downward, upper limit *T*(9) = – 14.9, lower limit *T*(9) = 12.0, all *p* < 0.001, Fig. 1C).

To determine measurement precision, the medians of SPV standard deviations per plane were computed: 0.34, 0.23, 0.34 and 0.30°/s at a sampling rate of 30 Hz for ConVNG and 0.11, 0.11, 0.12 and 0.12°/s at 220 Hz for VOG (all after ROUT at *Q* = 1%). Deriving an equivalence boundary of ± 0.12°/s from the maximum standard deviation of gold-standard VOG, TOST yielded significant results against all lower bounds (all p < 0.02), while comparisons against the upper bounds were not significant (Fig. 1D), meaning the data do not allow the conclusion of equivalence within the used interval.

In addition, Bayesian approaches to equivalence testing were used, which are outlined in detail in supplementary data.

### Retrospective validation in exemplary clinical cases

**Fig. 3:**
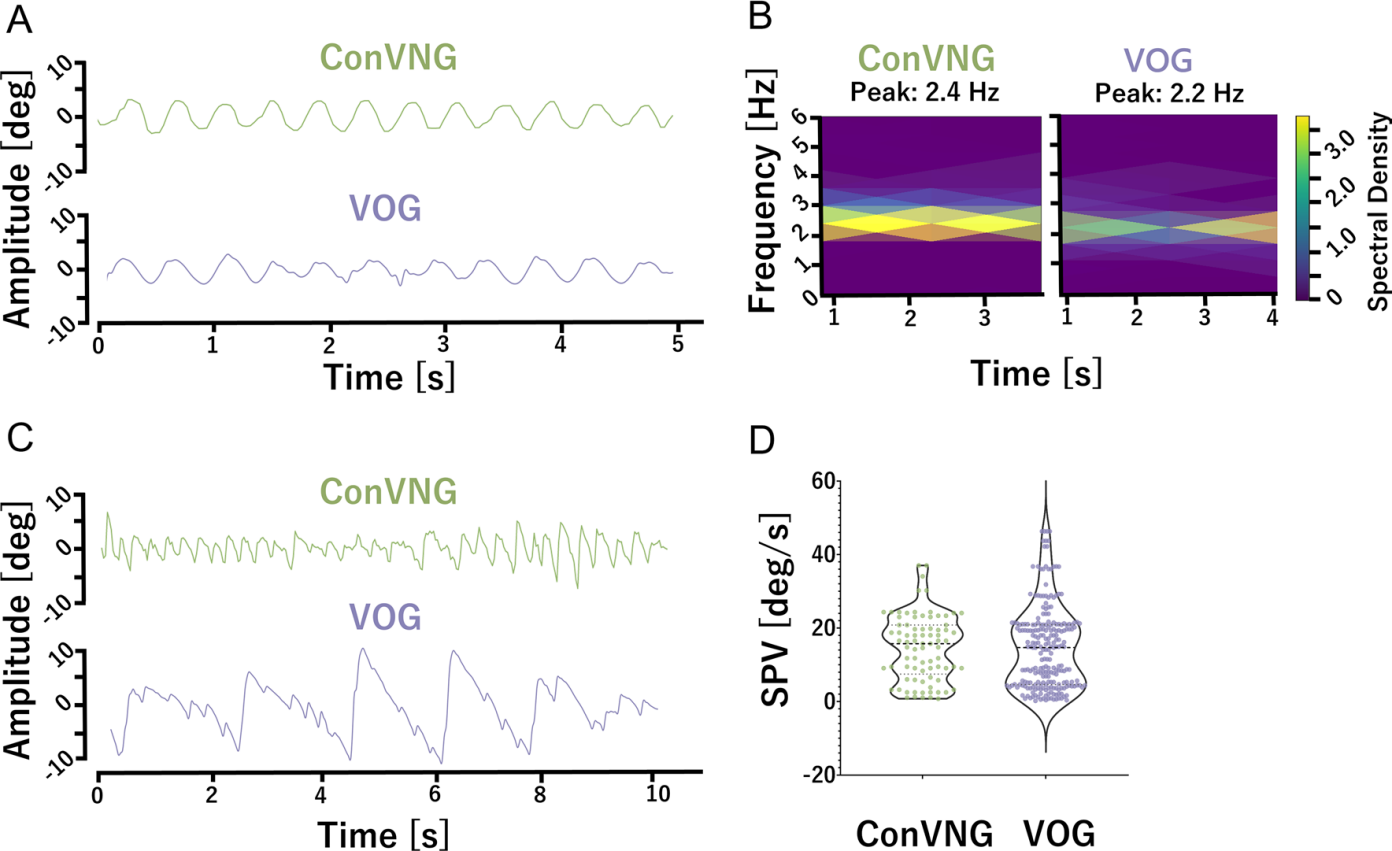
Retrospective validation. **A** Traces from gaze straight ahead in case 1 and **B** associated spectrograms. **C** Traces from case 2 and D., associated SPV calculations

In order to further assess the validity of ConVNG, the authors revisited recent jerk nystagmus cases from their respective clinics with both smartphone video documentation and VOG data available from the same examination to construct a retrospective convenience sample for validation. Likely due to the broad availability of VOG in the authors’ clinical settings, rendering simultaneous video documentation optional, only two cases were identified fulfilling the search criteria. The first subject presented with a pendular nystagmus restricted to the horizontal plane. Since SPV measurement is not fully applicable in pendular/sinusoidal nystagmus, frequency and amplitude relationships are used for evaluation, as their product ultimately influences visual acuity [1]. Since the nystagmus showed gaze-dependent shifts in intensity, selected signal portions with gaze straight ahead were used for comparison. Computed spectrograms revealed a peak frequency of 2.4 Hz (ConVNG) and 2.2 Hz (VOG), corresponding to an absolute deviation of 0.2 Hz (relative deviation 9%, Fig. 3A, B, supplementary video 5). The second subject showed a nystagmus purely beating in the vertical plane (downbeat nystagmus). Upward SPV measurements were 15.7 ± 8.9°/s (ConVNG) versus 14.9 ± 13.5°/s (VOG), constituting an absolute deviation of 0.8°/s (relative deviation 5%), a value falling well within the previously specified equivalence boundaries (Fig. 3C, D, supplementary video 6).

## Discussion

Quantitative analysis of eye movements is a quintessential technique in the clinical toolbox with immediate diagnostic implications, especially in the complex and highly interdisciplinary context of oculomotor disorders and vertigo management [10, 11]. Recent clinical investigations demonstrate that VOG-based assessments can double diagnostic certainty of vertigo syndromes in an emergency department setting [6] and shorten time to diagnosis in episodic vertigo syndromes which usually do not coincide with patient presentations [7]. Furthermore, videonystagmography offers the unique capability of demasking subtle oculomotor deficits and can also double the diagnostic yield of oculomotor assessment in the setting of ataxia syndromes, thereby providing crucial diagnostic clues guiding targeted genetic diagnostics [12]. However, the resource intensiveness of current gold-standard VOG precludes broad implementation, thereby demarcating factual and optimal management of neurological, otoneurological and neuroophthalmological patient populations.

To validate ConVNG, we chose a standardized, optokinetic instead of a caloric stimulus for two main reasons. First, caloric nystagmus is inter- and intraindividually highly variable due to unforeseeable central habituation mechanisms on various timescales [49, 62], making a ground truth estimation impossible. This is in contrast to well established normative data available for optokinetic nystagmus [59]. Second, caloric nystagmus is significantly suppressed by visual fixation, necessitating either Frenzel goggles, which introduce spatial distortions in video recordings or dark conditions, which demand infrared cameras not routinely usable in most consumer grade cameras. Both factors have likely contributed to the only modest agreement between webcam video- and gold-standard VOG-based nystagmus classification in a recent study by Reinhardt et al. [46] In another closely related study focusing on a continuous monitoring aspect, Phillips and colleagues have demonstrated how novel, wearable technologies enable nystagmus detection and classification [19]. However, the extraction of quantitative measurements like SPV, a quintessential descriptor of nystagmus pathophysiology with established diagnostic [6, 7] and therapeutic [9, 14] implications, has not been successful in both studies [19, 46]. In Young and colleagues’ study investigating a portable infrared VOG device as a vestibular event monitor, SPV measurements were not only acquired with high accuracy but could also differentiate common etiologies of episodic vertigo disorders [7]; however, a custom-built VOG device was needed.

The framework outlined here does not require any device except for a camera with at least 30 Hz temporal and 640 × 480 px spatial resolution, which is well below the current camera standard implemented widely in digital devices, webcams and the like. Making only few and pragmatic anthropomorphic assumptions regarding interpupillary distance and eyeball diameter [60, 61, 71], standard algorithms like filtering, fast Fourier transform, power spectral analysis, peak stripping and instantaneous gradient calculation (in line with standard SPV computations [72]) were used on the bilateral ConVNG-derived pupil centroid’s time series to identify nystagmus beating direction and calculate the average SPV per plane.

Despite a known bias of the TOST method towards non-equivalence decisions in small sample size scenarios like the one at hand [63], it revealed equivalence of both methods’ SPV measurements, which converged with the outcomes of additional, explorative Bayesian testing. In a benchmark comparison with MediaPipe, a state-of-the-art CV algorithm enabling both offline and real time, on device gaze estimation, ConVNG demonstrated superiority in terms of SPV ground truth equivalence and precision, while both algorithms yielded relative errors equivalent to VOG within the derived equivalence margins. These findings support the notion that CV algorithms like DLC and MediaPipe are not only capable of tracking pupils in laboratory acquired, physiological videos, but also of extracting time series data accurate enough to enable kinematic analyses in clinically relevant contexts. We speculate that ConVNG’s superiority in SPV calculation and precision partially stems from its domain specific fine tuning with clinical video material of eye movement pathologies, associated with ocular dynamics (misalignment, nystagmus) considerably deviating from physiological ocular variance distributions learnable from synthetic or laboratory data used for MediaPipe’s training [73]. Besides such eye movement alterations, clinical eye movement videos contain a myriad of facial obscurations (face masks, tubing, dressings, etc.) and are usually focused on the eye region. Since the study was conducted in a hospital setting during the pandemic, all prospective subjects wore face masks throughout their recordings. These additional sources of variance are likely to influence both gestalt and gaze tracking quality, a factor we specifically accounted for when annotating > 550 frames of diverse videos for ConVNG fine tuning. Our results demonstrated that both MediaPipe and even more so, OpenFace, ideally operate on video material with full and unobstructed face coverage, which might reduce their robustness for clinical eye movement analysis.

ConVNG was furthermore used to derive kinematic descriptors of retrospective clinical cases from our center. In both experimentally uncontrolled instances (handheld camera recording in case DBN), ConVNG-derived kinematic nystagmus descriptors with relative deviations in relation to VOG as little as 9% and 5%, respectively. Albeit the SPV extracted from retrospective case DBN, which was recorded with a handheld smartphone camera, closely matches VOG-derived SPV, the resulting pupil traces show waveform attenuating low frequency contamination due to relative camera movements. While slight improvements in signal preprocessing could improve this issue, the trace quality of case CN, recorded using a tripod, is considerably higher. Therefore, camera stabilization may be among the few important constraints for ConVNG in order to operate to its full potential. Applying ConVNG to randomly sampled instances from a fully independent dataset [44] depicting eye movement disorders in real-world clinical settings yielded excellent out-of-sample robustness (median pupil label likelihood > 0.85), suggesting feasibility of retrospective quantitative eye movement analysis regardless of experiment, setting or camera equipment.

This development adds to the growing interest in accessible, smart health care applications. As an important example, Parker and colleagues provided the proof-of-concept of an ARKit [38]-based iPhone application for video head-impulse testing [41, 42], thereby demonstrating feasibility of smartphone application-based eye and head movement recordings, albeit a considerable effort for manual postprocessing was needed. Using their ARKit-based application for gaze estimation, accuracy of 17% for horizontal and 27% for vertical planes (average 23%) and precision of 1.3° at lower gaze eccentricities was reported [42]. For comparison, precision of ConVNG reached 0.23–0.34°/s, MediaPipe 0.6–2.3°/s and, as expected, VOG reached precision as high as 0.11–0.12°/s. While equivalence of ConVNG, MediaPipe and VOG in regards to precision could not be demonstrated, ConVNG’s absolute precision of < 0.34°/s is almost one order of magnitude higher than ARKit’s documented gaze estimation [38], ~ 75% higher than the values achieved by Parker and colleagues [42] and at least twofold higher than MediaPipe.

Notably, unlike VOG, both comparable ARKit approaches and ConVNG do not implement a formal calibration procedure. Enabling the relation of absolute pupil and stimulus positions in 3D space, calibration is crucial for VOG tasks intended to yield gain value calculations (e.g., for video head-impulse testing or saccadometry) and alleviate image distortions introduced in eccentric gaze angles [30]. In comparison to head mounted VOG setups covering up to 40° of viewing angle, our experimental design covered ~ 20°, thereby leading to less eccentricity-related image distortions. In this context, an implicit calibration procedure consisting of two anthropomorphic assumptions and a stable viewing distance of approximately 40 cm as outlined above was sufficient to allow extraction of data with high accuracy. At the same time, the lack of calibration might partly explain why both ConVNG, MediaPipe and ARKit’s [38] precision metrics were clearly inferior to those of reference VOG. The possibility of calibration-free measurement with sufficient accuracy, however, is of particular relevance for at-home monitoring, especially in short-lasting, episodic conditions or frontline settings which may practically not allow lengthy calibration procedures and associated user input-intensive device interaction.

Among the main methodological limitations of this study is the need for sequential eye movement recordings since ConVNG is unable to track pupils and facial landmarks largely occluded or obscured by VOG goggles. However, in stark contrast to caloric nystagmus [46, 62], optokinetic nystagmus dynamics are significantly more stable between sequential measurements, making relevant intra-individual fluctuations confounding performance comparisons highly unlikely [1, 57]. In addition, we took precautions so as to avoid systematic biases by randomizing the order of experimental conditions for every individual. Still, this fact introduces an unforeseeable degree of biological variance, which needs to be considered when interpreting the equivalence testing and especially the comparisons of the retrospective cases. Notwithstanding, elimination of this variance causing experimental factor might rather lead to higher than lower accuracy and precision metrics.

Another important limitation of this study is the non-exhaustive representation of different nystagmus velocity conditions which would enable testing for correlations, linearity and systematical error distributions, but lie beyond the scope of this intended proof-of-concept. Instead, we defined ground truth values which closely match SPV values typically encountered in clinical practice to maximize validity [6, 7].

Relying on RGB videos, our approach is not expected to function in low light and low contrast settings, as was demonstrated in the out-of-sample validation. Combining the iPhone’s natural light and infrared sensors [38, 41], ARKit eye tracking promises to be more robust in these settings. However, in a series of experiments probing ARKit’s eye tracking capabilities (Taeger & Friedrich et al., unpublished data), we found that tracking performance significantly deteriorated in dim light conditions to the point of signal-to-noise ratios insufficient for SPV calculation. A recently proposed workflow based on convolutional neural networks applied to VOG-derived infrared eye videos has been associated with the most favorable pupil segmentation performance known to date [30], however, as opposed to ConVNG it requires VOG equipment (high resolution infrared eye videos) in the first place. Albeit highly relevant for applications requiring visual fixation suppression, broadly accessible and available infrared camera technology remains an unmet need in clinical and experimental practice.

Taken together, our findings demonstrate that a 30 Hz smartphone video can be sufficient for a specifically trained CNN to track pupils from clinically relevant video material, extract quantitative eye movement parameters with an accuracy comparable to VOG and a precision higher than comparable CV frameworks. Prospective, large-scale experiments in real-world clinical settings are warranted to validate robustness and reliability of ConVNG. To this end, we make the pretrained ConVNG model publicly available for the clinical and scientific community to build upon (https://doi.org/10.7910/DVN/GTUMAJ).

## Supplementary Information

Below is the link to the electronic supplementary material.

Supplementary file1 (DOCX 337 KB)

## Data Availability

Non-identifiable data and code sufficient to reproduce this study’s main claims are available upon request to the corresponding authors. The CNN model can be openly downloaded at the Harvard dataverse: https://dataverse.harvard.edu/dataset.xhtml?persistentId=doi:10.7910/DVN/GTUMAJ.
