# Supplementary material for: Smartphone video nystagmography using convolutional neural networks: ConVNG
Source: J Neurol. 2022 Nov 23;270(5):2518–30. doi: 10.1007/s00415-022-11493-1 (PMC10129923; doi:10.1007/s00415-022-11493-1)
Supplement: Supplementary file 1 — Supplementary file1 (DOCX 337 KB) [file 415_2022_11493_MOESM1_ESM.docx]

# Supplementary data

## Comparative model validation

In order to establish a benchmark comparison, we aimed to relate ConVNG’s pupil tracking performance to existing computer vision frameworks for human gaze estimation from videos, namely OpenFace^35^ and MediaPipe’s iris model^37,73^ (henceforth referred to as “MediaPipe”), both of which have been validated with large datasets taken in physiological contexts (e. g. a version of the Max Planck Institute for Informatics “Appearance-based gaze estimation in the wild”, MPIIGaze dataset^74^). The respective gaze tracking accuracies were reported to lie in the range of 7.9° to 13.91° for OpenFace^75^ and 7.14% mean absolute deviation normalized to the interpupillary distance for MediaPipe^73^. Recent investigations suggest that both algorithms might hold clinical utility for facial and appendicular movement analysis^39,40^.

### Prediction confidence

To compare model performance, ConVNG, OpenFace and MediaPipe were applied to the video datasets so as to derive each model’s prediction performance, expressed as the mean prediction confidence value and a fraction of tracked frames (FTF) per video. FTF reflects the amount of extracted data points constructing the pupil time series signal, thereby likely affecting downstream calculations of eye movement kinematics. While frame wise prediction confidence can be explored with OpenFace, MediaPipe only returns the fraction of frames tracked above a user defined confidence threshold.

Operationalizing sufficient frame wise prediction confidence with a threshold of ≥0.6 as previously described^45^, the fractions of tracked frames per video (FTF) were derived for all three algorithms applied to the prospectively collected video dataset used for SPV extraction. Friedman testing yielded significant differences (Friedman statistic= 62.97, p< .0001). Post-hoc testing with Dunn’s correction revealed that ConVNG showed a significantly higher FTF than OpenFace (0.26± 0.36 vs. 0.9± 0.14, p< .0001), while Mediapipe’s FTF was slightly higher than ConVNG (0.92± 0.21 vs. 0.90± 0.14, p= .001, supplementary figure 1A). Assessing the associated descriptive statistics in more detail showed that, while ConVNG performed robustly across all prospective videos (minimum FTF 0.54, range 0.46), both OpenFace and mediapipe failed to extract sufficient data points in some instances (minima 0.0 and 0.18, ranges 0.98 and 0.82, respectively).

ConVNG’s frame wise mean prediction confidence was significantly higher than OpenFace (0.99± 0.005 vs. 0.26± 0.33, supplementary figure 1B). Since in contrast to MediaPipe, OpenFace seemed to heavily rely on detection of a full-face appearance, we repeated the analyses with uncropped videos showing the whole face. While OpenFace’s prediction confidence significantly improved (p= .0002), it was still well below ConVNG’s mean prediction confidence (Friedman statistic 76.2, p< .0001 Dunn-corrected, supplementary figure 1B).

### SPV calculations

Given OpenFace’s relatively low pupil tracking confidence in our dataset, subsequent SPV comparisons were conducted only with time series extracted from MediaPipe. Both MediaPipe and DLC extract two-dimensional coordinate time series in identical fashion (x and y marker positions in pixel/ frame dimension), enabling direct and meaningful comparisons. To this end, SPVs were computed using the presented postprocessing protocol with one modification: since we observed that determination of nystagmus direction (using FFT and subsequent gradient detection) failed in 8/40 time series derived from MediaPipe, partly due to large label fluctuations and missing or implausible marker predictions, we replaced FFT with a similar wavelet transform on the whole time series, ultimately enabling detection of respective power maxima in 39/40 time series to enable meaningful comparisons. No other parameters were changed.

Plane wise comparisons of ConVNG and MediaPipe extracted SPVs revealed significant differences (leftward, p= .006, rightward, p= .014). Paired TOST analyses comparing MediaPipe and VOG-derived SPVs failed to prove equivalence within clinically derived boundaries of ±2.5°/s in 5 out of 8 comparisons (significant comparisons: upward, upper limit T(9)= -3.1, p= .0006, lower limit T(9)= 3.34, p= .004; downward, lower limit T(9), p= .037, supplementary figure 1C-D). For horizontal directional symmetry, TOST revealed equivalence in one comparison (lower limit T(9)= -3.03, p= .001).

Relative errors of MediaPipe derived SPVs were calculated to be leftward 25.3± 24.8%, rightward 20.8± 25.4%, upward 5.6± 16.2% and downward 19.5± 34.5% (ConVNG: leftward 11.8± 14.4, rightward 9.5± 15.3, upward 11.4± 7.3, downward 8.9± 6.3%, supplementary figure 1E-F). No statistical difference of ConVNG and MediaPipe derived SPV relative errors to ground truth could be detected. Within the equivalence boundary of ±38.9%, MediaPipe’s SPV relative errors were equivalent to VOG (leftward, upper limit T(9)= -5.74, p< .001, lower limit T(9)= 3.41, p= .0004; rightward upper limit T(9)= -4.21, p= .001, lower limit T(9)= 3.6, p= .003; upward, upper limit T(9)= -4.6, p< .001, lower limit T(9)= 7.7, p< .001; downward, upper limit T(9)= -4.5, p< .001, lower limit T(9)= 3.16, p= .006, supplementary figure 1E-F).

The medians of MediaPipe derived SPV standard deviations per plane were computed to be 0.6, 2.3, 0.75 and 0.65°/s (median 0.7°s; ConVNG 0.34, 0.23, 0.34 and 0.30°/s, median 0.3°/s). Plane wise comparisons demonstrated significantly higher precision of ConVNG in the vertical plane (upward p= .004, downward p= .005); in one horizontal plane, comparison approached statistical significance (rightward p= .055, supplementary figure 1G-H). Implementing an equivalence criterion of 0.12°/s for precision as derived from the maximum standard deviation of goldstandard VOG, TOST demonstrated non-equivalence of MediaPipe SPV precision in comparison to VOG (all upper bounds p> .5, supplementary figure 1G-H).

To exclude a potentially negative influence of low confidence predictions on SPV calculations, we repeated analyses restricted to data points with a confidence threshold set at 0.6, aligning with previous work^45^.

Paired TOST analyses comparing MediaPipe’s and VOG-derived SPVs failed to prove equivalence within clinically derived boundaries of ±2.5°/s in 5 out of 8 comparisons (leftward, lower limit T(9)= 2.19, p= .028; upward, upper limit T(9)= -3.35, p= .004; downward, upper limit T(9)= -2.2, p= .027, all remaining comparisons p> .5). For horizontal directional symmetry, TOST revealed equivalence (upper limit T(8)= -3.03, p= .008, lower limit T(9)= 4.05, p= .002).

Relative errors of MediaPipe derived SPVs were calculated to be leftward 35.1± 36.8%, rightward 45.6± 46.2%, upward 42.3± 50.8% and downward 52.5± 52.0%. Nominally, mean relative errors were omnidirectionally larger than ConVNG (leftward 11.8± 14.4, rightward 9.5± 15.3, upward 11.4± 7.3, downward 8.9± 6.3%), which was statistically significant in the rightward condition (p= .037). Moreover, mean error derived from thresholded versus unthresholded time series signals was significantly larger in rightward direction (p= .037). In an equivalence boundary of ±38.9%, MediaPipe’s thresholded SPV relative errors were equivalent to VOG in 4 of 8 comparisons (leftward, upper limit T(9)= -4.5, p< .001; rightward, upper limit T(8)= -4.45, p= .002; upward, upper limit T(9)= -4.3, p< .001; downward, upper limit T(9)= -4.73, p< .001).

The medians of MediaPipe derived SPV standard deviations per plane were computed to be 0.6, 1.0, 0.5 and 0.52°/s; plane wise comparisons demonstrated significantly higher precision of ConVNG in all but the leftward comparisons (rightward p= .02, upward p= .009, downward p= .013). Within the equivalence boundary of 0.12°/s derived from the maximum standard deviation of goldstandard VOG, TOST demonstrated non-equivalence of MediaPipe SPV precision in comparison to VOG (all upper bounds p> .5). No significant differences could be detected when comparing precision from thresholded versus unthresholded MediaPipe time series.

Taken together, MediaPipe’s FTF was found to be slightly higher than ConVNG, which of course does not allow any inferences as to the actual frame wise accuracy of pupil tracking (reported to be ~7%, related to interpupillary distance^73^, ConVNG’s accuracy: 9-15% related to pupil diameter). MediaPipe extracted time series’ lower signal to noise ratio negatively affected nystagmus detection, requiring slight algorithmic optimization. Regardless of prediction confidence thresholding, MediaPipe derived SPVs were not found to be equivalent to gold standard in the majority of comparisons, in contrast to ConVNG derived SPVs, which were clinically equivalent in all comparisons. Accuracy of SPV was not statistically different between MediaPipe and ConVNG and reached equivalence to gold standard. ConVNG however achieved significantly higher precision than MediaPipe in the vertical plane. MediaPipe precision was not equivalent to VOG in all planes. Repeating comparisons with frame wise prediction confidence thresholding at 0.6 did not significantly alter these results.


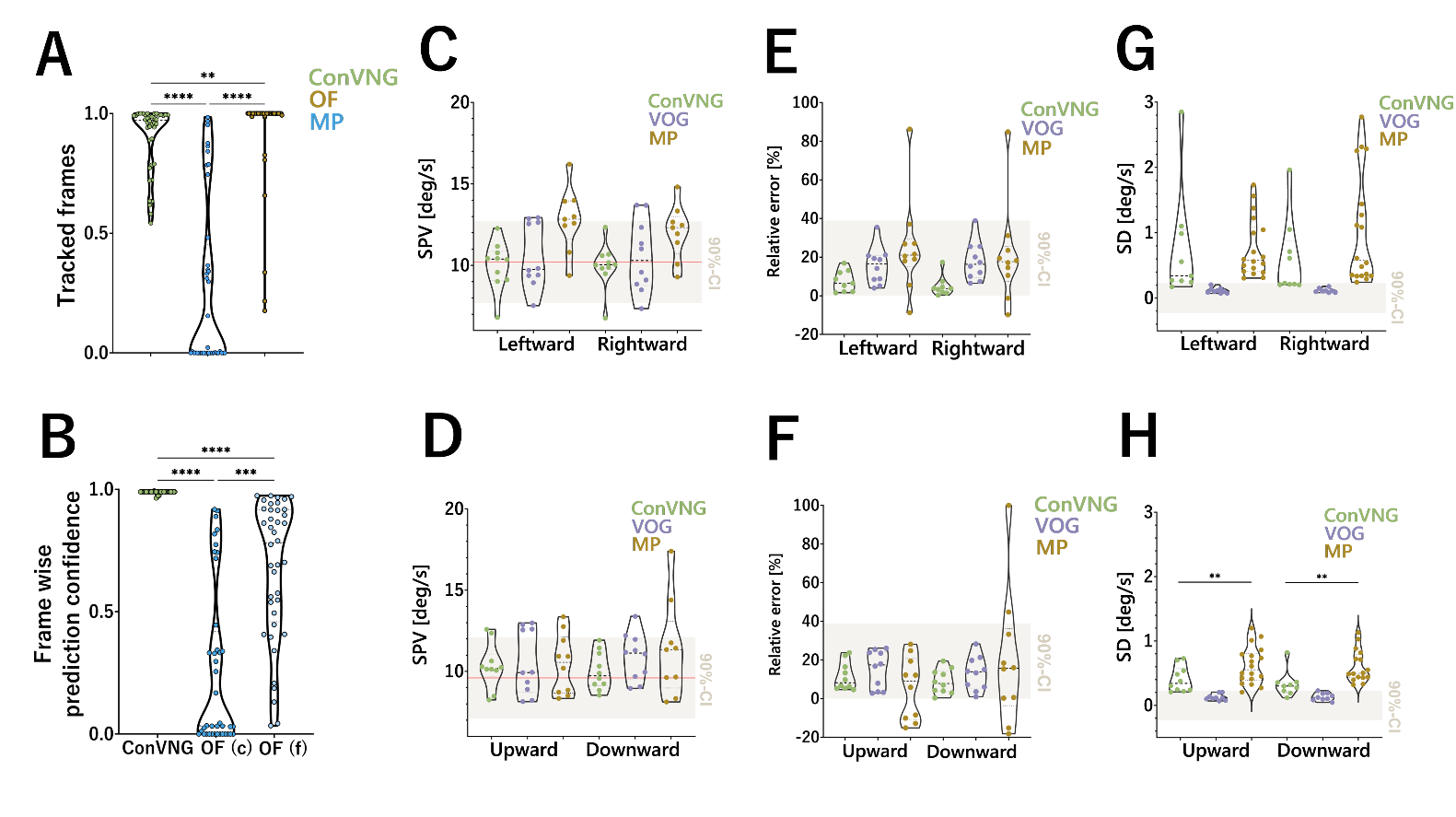


**Supplementary figure 1**. **A.** Comparison of frames tracked above a frame wise confidence threshold of 0.6; OF= OpenFace, MP= MediaPipe. **B.** OF mean frame wise confidence significantly increases in full face versus videos cropped to the eye region but is significantly lower than ConVNG. C., D., Equivalence testing of SPV estimates. E., F., Equivalence testing of accuracies and G., H. of precision metrics. Of note, ConVNG shows significantly higher precision in both vertical planes than MP (p< .01).

## Explorative equivalence analysis using Bayes interval-null method

In light of its potential complimentary value in small sample and large effect size settings like the one at hand^63,76,77^, a more explorative approach to equivalence testing using Bayesian statistics was applied to this work’s key finding, i. e. SPV accuracy and precision. To this end, the smallest (i. e. most conservative) effect sizes, expressed as Cohen’s d values, corresponding to the raw interval boundaries (±2.5°/s) used for TOST were used to delineate a maximum equivalence interval around nil^77^. Systematic exploration of the equivalence likelihood was conducted in a bipartite approach: first, the maximum d values were multiplied by 0.5 and 0.1 so as to map out likelihoods of equivalence as a function of increasingly conservative interval boundaries. Second, due to insufficient data to inform a prior, a default Cauchy prior centered at 0 with a scale of $\frac{1}{\surd2}$ ≈ 0.707 was used besides $\frac{0.5}{\surd2}$ ≈ 0.354 and $\frac{2}{\surd2}$ ≈ 1.41 as more skeptical or “optimistic” variants, in line with previous comparative and explorative modeling in equivalence testing^63,78^.

In the Bayes interval-null method, the so called non-overlapping hypothesis (NOH) Bayes factor (BF) compares the likelihoods of the interval-null hypothesis (i. e. measurements are equivalent, H_0_) and the alternative hypothesis (i. e. effect sizes fall outside the equivalence region, suggesting non-equivalence, H_1_). NOH BF can be viewed as the degree, to which the data support values lying within versus outside of the equivalence intervals. The overlapping hypothesis (OH) BF compares the interval-null hypothesis against an unconstrained (i. e. no equivalence region around nil) alternative hypothesis and can be interpreted as the degree to which the data support the use of a specified equivalence region^77^.

Using an equivalence region of d ± 1.02 (smallest of all d-values corresponding to ±2.5°/s raw SPV interval boundaries used in TOST comparisons), OH BF for SPV comparisons per plane were 1.64, 1.64, 1.64 and 1.60, NOH Bayes factors 193.33, 235.65, 749.28 and 24.61, providing weak evidence for the overlapping hypothesis (i. e. restriction of parameter space to d ± 1.02) and extremely strong evidence in favor of equivalence. In other words, there is weak evidence in favor of the comparatively large equivalence boundary and given the data, the equivalence hypothesis is at least ~25 times as likely as the non-equivalence hypothesis. At d ± 0.5, OH Bayes factors were 2.55, 2.64, 3.07, 1.40 and NOH Bayes factors were 3.0, 3.15, 3.9 and 1.46. At d ± 0.1, OH Bayes factors were 2.55, 2.64, 3.07 and 1.40, NOH Bayes factors were 3.0, 3.15, 3.85 and 1.46. Overall, these values provide moderate to strong evidence for an equivalence boundary of at least d ± 0.5, within which the equivalence hypothesis is ~1.5- to 3.9-fold more likely than the alternative hypothesis. Neither adjusting priors to more skeptical $(\frac{0.5}{\surd2}$ ≈ 0.354) nor optimistic ($\frac{2}{\surd2}$ ≈ 1.41) values substantially changes this conclusion (see supplementary figure 2 for systematic illustration).

For relative error equivalence testing, OH Bayes factors were 1.32 in all planes and NOH Bayes factors >142.285, providing weak evidence for the overlapping hypothesis and very strong evidence in favor of the equivalence hypothesis. At $\frac{dmax}{2}$ = ± 0.89, OH Bayes factors were 1.71, 1.71, 1.72 and 1.70 and NOH Bayes factors 34,67, 33.51, 45.21 and 25.1. At $\frac{dmax}{10}$ = ± 0.18, OH Bayes factors were 2.04, 2.02, 2.20 and 1.84, NOH Bayes factors were 2.53, 2.50, 2.83 and 2.20. Overall, this provides moderate to strong evidence in favor of the equivalence hypothesis within the smallest boundaries, d ± 0.18. As was the case for SPV measurements, adjusting priors to more skeptical $(\frac{0.5}{\surd2}$ ≈ 0.354) or optimistic ($\frac{2}{\surd2}$ ≈ 1.41) values yielded expected shifts of Bayes factors, however not substantially changing the conclusions drawn using the default prior (data not shown).

For precision estimates (d ± 0.14, corresponding to 0.12°/s minimal precision of VOG used in TOST), OH BF were 0.97, 1.04, 0.74 and 0.95, NOH BF were 0.97, 0.96, 0.72 and 0.95. At d ± 0.07 OH Bayes factors were 0.94, 1.0, 0.70, 0.92 and NOH BF were 0.93, 1.0, 0.69 and 0.91. At d ± 0.014 OH BF were 0.92, 0.99, 0.69, 0.90 and NOH BF were 0.92, 0.99, 0.69, 0.90. With NOH Bayes factors ranging between 0.69 and 1.0, consistent with weak evidence against the equivalence hypothesis with largely insufficient data to support a definitive conclusion.

Taken together, the Bayesian interval-null method’s outcomes across exploratory parametrizations of priors and equivalence region boundaries stand strongly in favor of the equivalence hypothesis in terms of clinically relevant accuracy intervals while providing largely inconclusive evidence for definitive conclusions for precision comparisons. Overall, these findings closely converge with TOST findings, thereby corroborating the equivalence hypothesis.


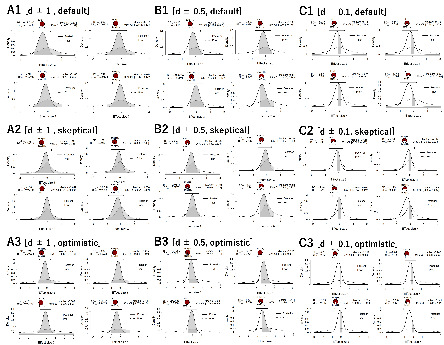


**Supplementary figure 2** - SPV equivalence testing using Bayes interval null method. Shown are the relationships of prior (dashed lines) and posterior (solid lines) likelihood distributions with equivalence regions shaded in grey. Rows (i. e. A-C) ordered by d-values for equivalence boundaries, columns (i. e. 1-3) ordered by prior estimates (see supplementary methods).

## Supplementary videos

| **ID** | **Content** |
| --- | --- |
| 1-4 | Exemplary labeled videos of prospective cohort subjects showing nystagmus in all four planes. |
| 5 | Labeled video of Case 1, downbeat nystagmus |
| 6 | Labeled video of Case 2, pendular nystagmus |
| “OOSV”-Videos | Labeled videos randomly sampled from Dan Gold collection^44^ used for out-of-sample validation. |

**Supplementary Table 1** - Video descriptions.
